# The role of Gene Mutations (*gyrA, parC*) in Resistance to Ciprofloxacin in Clinical Isolates of *Pseudomonas Aeruginosa*

**DOI:** 10.30699/IJP.2021.520570.2542

**Published:** 2021-07-06

**Authors:** Nasibeh Arabameri, Zoheir Heshmatipour, Shima Eftekhar Ardebili, Zeinab Jafari Bidhendi

**Affiliations:** 1 *Department of Microbiology, Faculty of Science, Tonekabon Branch, Islamic Azad University, Tonekabon, Iran*

**Keywords:** Ciprofloxacin, gyrA, Mutation, parC, Pseudomonas aeruginosa

## Abstract

**Background & Objective:**

*Pseudomonas aeruginosa* is an opportunistic pathogen and one of the most common causes of nosocomial infections. This bacterium's antibiotic resistance to the common fluoroquinolone antibiotics, especially ciprofloxacin, is due to mutations in the *gyrA* and *parC* genes. This study aimed to investigate the effect of the mutation in (*gyrA*, *parC*) on ciprofloxacin resistance in clinical isolates of *Pseudomonas aeruginosa*.

**Methods:**

A total of 140 clinical samples were collected from hospitals. The samples were identified by standard biochemical tests, and the antibiotic resistance was investigated by the disk diffusion method. DNA was extracted from 30 isolates, and PCR was performed. PCR-sequencing was carried out to assess *gyrA* and *parC* mutations in drug-resistant isolates. NCBI-Blast and MEGA7 software was used to analyze the nucleotide sequences.

**Results:**

30 clinical isolates were 80% resistant to ciprofloxacin; meanwhile, in 21 samples, mutations were observed. 87/5% of mutations were related to *gyrA* (Thr83 → Ile), 79/16 % *parC* (Ser87 → Leu), and 4/18% (Glu91 → Lys). The antibiotic resistance to ciprofloxacin and mutations in *gyrA* and *parC* genes in resistant isolates are significantly related to each other (*P*<0.05).

**Conclusion:**

The mutations in the *gyrA* and *parC* genes play an essential role in resistance to ciprofloxacin in clinical isolates of *Pseudomonas aeruginosa*.

## Introduction

*Pseudomonas aeruginosa*, a genus Gammaproteo-bacteria, belongs to the large family of *Pseudomonas *(1, 2). In the form of biofilm, this bacterium has higher pathogenicity than planktonic (3). *P. aeruginosa* is the cause of 10% of common nosocomial infections (4).

Quinolones are broad-spectrum oral antibacterial agents that are widely used in therapy. They are lightweight hydrophilic molecules that inhibit DNA replication without affecting RNA or protein synthesis in susceptible bacteria. Quinolones include four gene-rations, of which ciprofloxacin is the second one (5, 6). The bactericidal effect of ciprofloxacin on resistant strains of *P. aeruginosa* is much more significant than other types of antibiotics that seem to treat *pseudo-monas-related* infections (7, 8). Fluoroquinolones can easily enter cells through purines, often used to treat intracellular pathogens such as *Legionella* *pneumo-phila* and *Mycoplasma pneumoniae*. 

In gram-negative bacteria, DNA gyrase, and gram-positive bacteria, topoisomerase IV is targeted (9, 10). The resistance of *P. aeruginosa* to fluoroquinolones, including ciprofloxacin, can be mediated by mutations in DNA gyrase and topoisomerase IV, reducing wall permeability and increasing efflux pump expression (11-14). DNA gyrase and topoisomerase IV are tetrameric enzymes with different subunits, *gyrA*, and *parC* from DNA gyrase homologous and, *parC* and *parE* from topoisomerase IV (15, 16). Genetic, biochemical, and epidemiological studies show that DNA gyrase is the first target, and topoisomerase IV is the second target of fluoroquinolones (17, 18). Mutations in the fluoroquinolone resistance determinant region (QR-DR) in the *gyrA* and *parC* genes are the major causes of fluoroquinolone resistance in *P. aeruginosa *(19, 20). This study's purpose is to investigate the role of mutations in *gyrA* and *parC* genes in the development of ciprofloxacin resistance in clinical isolates.

## Material and Methods

**Sample Preparation and Identification**

In 2019, 140 *P. aeruginosa* strains were collected as cross-section individuals from patients with cystic fibrosis, urinary infections, and diabetic wounds from Imam Khomeini Hospital, Resalat Hospital, and Bouali Hospital in Tehran, Iran. Bacteria were identified from various samples, including urine, blood, wounds, pleura, CSF, and characterized using various biochemical tests, including TSI reaction, OF test, Oxidase test, Catalase test, and Sim-on citrate test following the reference protocols (19, 21). The approved isolates were stored in a -70°C freezer for further testing. All tests were done in a microbiology laboratory in Islamic Azad University, Tonekabon, Mazandaran, Iran.

**Antimicrobial Susceptibility**

For further molecular studies, we initially selected all ciprofloxacin-resistant strains to ensure that the strains contain the potential resistance genes. Strains were incubated in LB broth overnight at 37°C. Using antibiotic disks (CONDA, Spain), include Rifampin, Trimethoprim-sulfamethoxazole, Ampicillin-sulbactam, Ciprofloxacin, Ceftriaxone, Amikacin, Imipenem, Gentamicin, Piperacillin-tazobactam, and Ceftazidime. Antibiotic resistance was determined based on the resistance patterns of the isolates by the disk diffusion method. A concentration of 1.5x10^8 CFU/mL of each overnight fresh culture was made individually, and an amount of 100μL were spread on Mueller-Hinton agar plates using sterile cotton swabs. Disks were placed on each plate with distinct space between to observe the inhibitory zones and incubated for another 24 h in 37°C. 

**Molecular Assay**

The GeneMarkbio product kit extracted the DNA of 30 ciprofloxacin-resistant isolates from Taiwan. The primer sequence was examined at the NCBI website using Blast software and subsequently produced by the Danish company (Tag-Copenhagen, Denmark). The sequence and size of the primer are mentioned in Table 1 (22). Following this, PCR was performed on 30 isolates and a negative control for possible contaminations. Following that, the PCR samples were sent to BIONEER, South Korea for sequencing, and then the results were analyzed using MEGA software and NCBI. The gyrA and parC genes sequence were compared with associated locus in *Pseudo-monas aeruginosa PAO1* AE004091 wild strain from NCBI GenBank as reference. Finally, the gene mutations that cause changes in the nucleotide sequence, including deletion or insertion of nucleotides, resulting in a shift in the amino acid sequence, were analyzed by MEGA7 software.

**Table 1:** Primers used in this study

Gene name	Nucleotide sequences	Band size	Reference
*gyrA*-F	5΄-GAC GGC CTG AAG CCG GTG CAC-3΄	460 bp	(22)
*gyrA*-R	5΄-GCC CAC GGC GAT ACC GCT GGA-3΄		
*parC*-F	5΄-CAT CGT CTA CGC CAT GAG-3΄	270 bp	
*parC*-R	5΄-AGC AGC ACC TCG GAA TAG-3΄		

## Results

**Antibiotic Susceptibility**

**Fig. 1:**
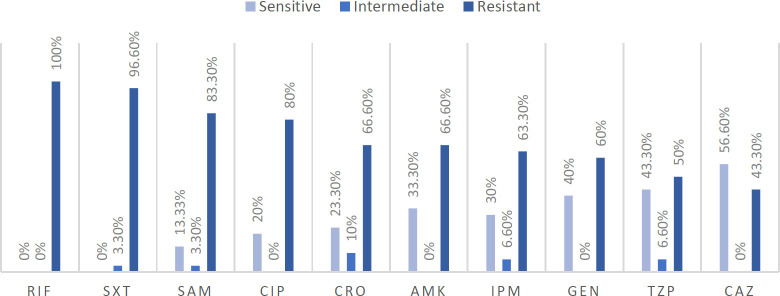
Results of *Pseudomonas aeruginosa* antibiotic resistance using Disk Diffusion Antibiotic Sensitivity test (The Kirby-Bauer test); abbreviations refer to rifampin (RIF), trimethoprim-sulfamethoxazole (SXT), ampicillin-sulbactam (SAM), ciprofloxacin (CIP), ceftriaxone (CRO), amikacin (AMK), imipenem (IPM), gentamicin (GEN), piperacillin-tazobactam (TZP), ceftazidime (CAZ).

The antibiotic susceptibility pattern in 30 samples out of 140, which are confirmed to be ciprofloxacin-resistant to *P. aeruginosa* using biochemical identifiers and disk diffusion assay, in random infected patients gathered from several hospitals in Tehran, as we mentioned before, showed different antibiotic resist-ance percentages and patterns, as shown in Figure 1. Strains were collected mostly from cystic fibrosis lungs, blood, urine, and wound samples of patients regardless of their age, medical history, or gender.

**Gene Screening Results**

**Fig. 2:**
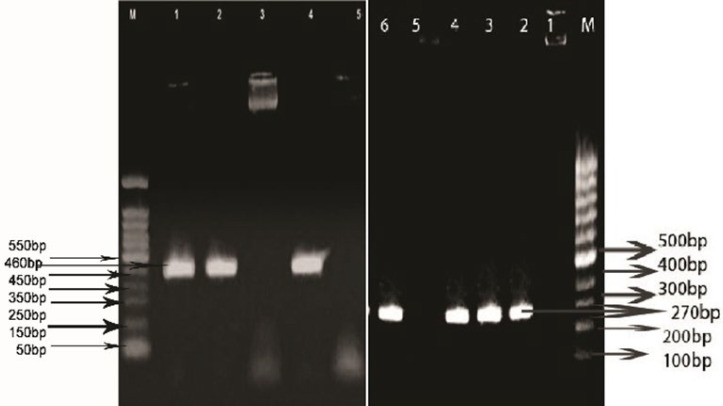
Results of electrophoresis of PCR products carrying *parC* and *gyrA* genes on 3% agarose gel

According to the results obtained from PCR products of 30 clinical samples, as shown in Figure 2, the product size of *gyrA* is 460 bp, and *parC* is 270 bp.

Findings of frequency percentages were analyzed by descriptive statistical methods such as Chi-Square, considering that the P-value from the frequency of antibiotic resistance and susceptibility to ciprofloxacin and mutations in *gyrA* and *parC* genes (0.023) is less than 0.05. Therefore, there is a significant relationship between ciprofloxacin antibiotic resistance and mutations in *gyrA* and *parC* genes.

**Fig. 3:**
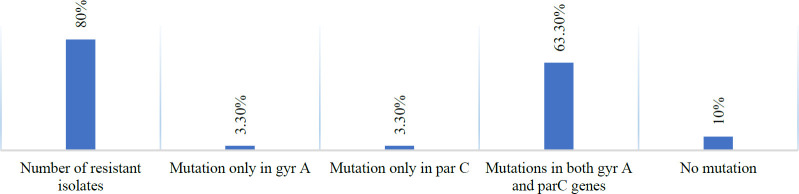
Ciprofloxacin resistance and gene mutation percentages in *P. aeruginosa* isolates

**Fig. 4:**
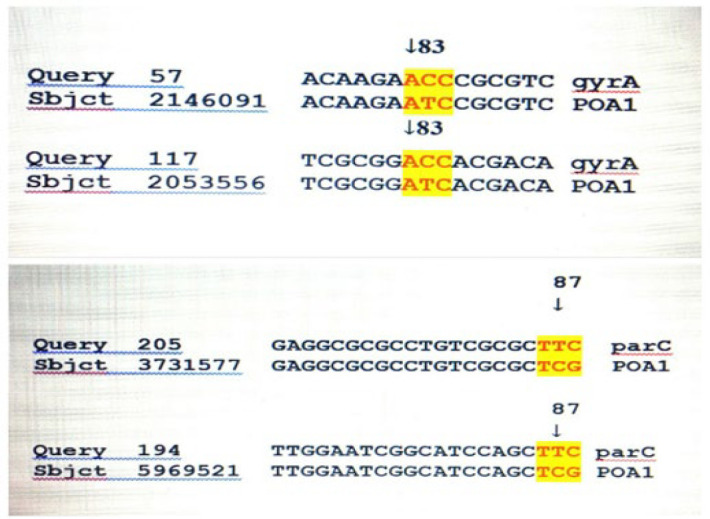
Sequence elements of *gyrA* and *parC* gene nucleotides

The point mutations in 24 ciprofloxacin-resistant isolates are as follow: 87.5% of mutations in codon 83 *gyrA* gene (Thr83→ Ile), 79.16% in codon 87 *parC* gene (Ser87 → Leu), and 4.18% in codon 91 *parC* gene (Glu91→Lys). Association between antibiotic susceptibility and mutations in the *gyrA* and *parC* genes in 30 clinical isolates is shown in Table 3 and Figure 3. These mutations are caused by the mismatch of nucleotides in a location of two strands (23), as shown in Figure 4. Besides, no mutations were observed in any of the sensitive isolates. The number and location of mutations in *gyrA* (Thr83 → Ile) and *parC* (Ser87 → Leu) genes were the same in 6 blood samples, 11 urine samples, and three tracheal samples. While in 1 isolate from the wound sample, a mutation in the *parC* gene was confirmed in codon 91 (Glu91→lys) and codon 87 (Ser87 → Leu), as shown in Table 3.

**Table 2:** The connection between ciprofloxacin sensitivity and mutations in *gyrA* and *parC* in 30 clinical specimens of *Pseudomonas aeruginosa*

	R(Resistant)	I(Intermediate)	S(Sensitive)	Total
Number of isolates	**24**	**0**	**6**	**30**
Mutation only in *gyr A*	**1**	**0**	**0**	**1**
Mutation only *par C*	**1**	**0**	**0**	**1**
Mutations in both gyr A and *parC* genes	**19**	**0**	**0**	**19**
No mutation	**3**	**0**	**6**	**9**

**Table 3:** Types and positions of *gyrA* and *parC* mutations in Pseudomonas aeruginosa clinical specimens

Amino acid changes	Mutation	codon	Number	Gene
Thr-Ile	ACC-ATC	83	21	*gyrA*
Ser-Leu Glu-Lys	TCG-TTG GAG-AAG	87 91	19 1	*parC*

## Discussion

*Pseudomonas aeruginosa* is the most common pathogen in the genus *Pseudomonas *and is the third most common nosocomial infection after *Staphylo-coccus aureus* and *Escherichia coli *(13, 24). This opportunistic pathogen has acquired high resistance to antibiotics, causing various infections, and is responsible for one of the deadliest sepsis among gram-negative bacteria by entering the bloodstream (25-27).

In recent years, extensive use of antibiotics causes resistance to broad-spectrum antibiotics among pathogenic bacteria. Multidrug-resist-ant strains (MDR) are currently the main problem in treating nosocomial infections in different hospital wards, such as burn centers or intensive care (28-30). The mechanism of action of ciprofloxacin is inhibition of DNA gyrase and topoisomerase IV. DNA gyrase is the bacterial topoisomerase II that controls the topology of the double helix of DNA during the replication and translation process (31-33).

However, several cases of resistance of *Pseudo-monas* isolates to this group of antibiotics have been reported. The most important mechanism of bacterial resistance to fluoroquinolones is the mutation in genes encoding DNA gyrase (*gyrA*) and topoisomerase IV (*parC*) (34).

*P. aeruginosa* antibiotic resistance has been the subject of many studies with different results regarding the surveys' time and geographical location. The antibiotic resistance ratio of the current research is as follow: rifampin 100%, cotrimoxazole 96.6%, ampi-cillin sulbactam 83.3%, ciprofloxacin 80%, ceftriaxone 66.6%, amikacin 66.6%, imipenem 63.3%, gentamicin 60%, Piperacillin tazobactam 50%, and ceftazidime 43.3%. In contrast, a study supervised by Ekrami and Kalantar in 2007 on 182 strains of *P. aeruginosa* isolated from burn patients showed 100% resistance to ciprofloxacin, gentamicin, amikacin, tobramycin, and ceftazidime,(35) which differs from the present study; this may be due to differences in the time and type of isolations. In 2007, Saderi* et al.* examined 186 isolated samples and reported 74.2% resistance to ceftazidime, 38.2% to imipenem, and 49.2% to ciprofloxacin(6). However, in the present study, ciprofloxacin resistance is 30% greater. It could be due to differences in the annual use of antibiotics and different physicians' diagnoses in timely treating infection.

In studies done by Ziyuan Yang* et al.* in southern China in 2015 (36) and Nouri* et al.* in Tabriz in 2016 (37) on the clinical isolation of ciprofloxacin-resistant *P. aeruginosa*, mutations in codon 83 of the *gyrA* gene, which converts the amino acid threonine to isoleucine, and mutations in the codon 87 of the *parC* gene, which changes the amino acid serine to leucine, played a significant role in resistance to ciprofloxacin. In addition to the above mutations, another mutation in the *parC* gene changes the amino acid glutamine to lysine in the present study. The findings are almost identical, and this slight difference may be related to sample collection and time variability.

In another study in Japan by Kobayashi* et al.* (2013), the cause of fluoroquinolone resistance in clinical strains of *P. aeruginosa* was caused by mutations in the *gyrA* (Thr83 → Ile), *parC* (Ser87 → Leu) genes, and overexpression of the efflux pump. This research on mutations in *gyrA* and *parC* genes is the same as the present study (38); however, there is a noticeable difference in antibiotic resistance to Cipro-floxacin, which may be due to geographical differences and arbitrary use of antibiotics in our country.

In Denmark (2000), Shah Jalal* et al.* researched on fluoroquinolone-resistant isolates found that the efflux pump is more effective than studied genes in terms of developing resistance in isolates from cystic fibrosis patients (33). With few studies in other countries, it has been shown that in strains isolated from cystic fibrosis patients, mutations in the *gyrA* and *parC* genes play a less important role than efflux pumps in fluoroq-uinolone resistance (39).

In Lebanon in 2013, Selma* et al.* reported 19 mutations in the *gyrA* and *parC* genes in ciprofloxacin-resistant *P. aeruginosa *strains (40). In Bulgaria in 2014, Estavov* et al.* identified mutations in *gyrA*, *parC*, and *MexR*, which were in the *gyrA* gene at codon 83, in the *parC* gene codons 87 and 136, and the *MexR* gene at codons 126 and 44. Finally, they realized that mutations in the *gyrA* gene were present in all ciprofloxacin-resistant strains of *P. aeruginosa *(41, 42). In this mutation, converting the polar amino acid threonine to a non-polar amino acid and hydrophobic isoleucine *gyrA* (Thr83 → Ile), the structure of DNA gyrase is altered, resulting in a decrease in the enzyme's tendency to react with antibiotics, eventually leading to drug resistance (18). Several studies have been performed on the *gyrA* gene, resulting in mutations in codons 83 and 87, including (Thr83 → Ile), (Asp87 → Asn) and (Asp87 → Tyr) (10, 26). While in the present study, only one mutation was observed in *gyrA* because of the dissimilarity in the type of clinical samples, time, and geographical area. 

## Conclusion

Based on the data obtained from other studies and present research, it was established that *Pseudomonas aeruginosa*, due to its genetic, is potentially receptive to a variety of genes such as transposons and plasmids, and therefore can quickly become resistant to anti-biotics. Thereby, due to this organism's capabilities in acquiring resistance to various antibiotics, continuous monitoring of changes in bacterial susceptibility is ess-ential. Improper use of antibiotics, especially fluoro-quinolones, is a risk factor for this bacterium's resis-tance to medicine in Iran. Because *P. aeruginosa* is an opportunistic pathogen in hospital settings, these isolates should be detected in clinical laboratories to provide appropriate treatment for infections to prevent their spread. Regarding two genes encoding *gyrA* and *parC* that were investigated in this study, further studies should be performed on other possible genes that are involved in the development of ciprofloxacin resistance in order to achieve more comprehensive and complete results, and find better outcomes comparing phenotypic and genotypic methods.
